# Supplementary material for: Study of key residues in MERS-CoV and SARS-CoV-2 main proteases for resistance against clinically applied inhibitors nirmatrelvir and ensitrelvir
Source: Npj Viruses. 2024 Jun 24;2:23. doi: 10.1038/s44298-024-00028-2 (PMC11196219; doi:10.1038/s44298-024-00028-2)
Supplement: Supplementary file 1 — Supplementary Materials [file 44298_2024_28_MOESM1_ESM.pdf]

Supplementary Materials for

Study of key residues in MERS-CoV and SARS-CoV-2 main proteases for resistance against clinically applied inhibitors nirmatrelvir and ensitrelvir

Laura Krismer, Helge Schöppe[≙], Stefanie Rauch[≙], David Bante, Bernhard Sprenger, Andreas Naschberger, Francesco Costacurta, Anna Fürst, Anna Sauerwein, Bernhard Rupp, Teresa Kaserer, Dorothee von Laer, Emmanuel Heilmann

Correspondence to: emmanuel.heilmann@i-med.ac.at

This PDF file includes:

Supplementary Figures 1 to 6
Tables 1 to 4
Captions for Alignments 1 to 7
Captions for Plasmids 1 to 7
Captions for Sequences 1 to 8
Captions for Files 1 to 3

Other Supplementary Materials for this manuscript include the following:

Alignments 1 to 7
Plasmids 1 to 7
Sequences 1 to 8
Files 1 to 3

Supplementary material

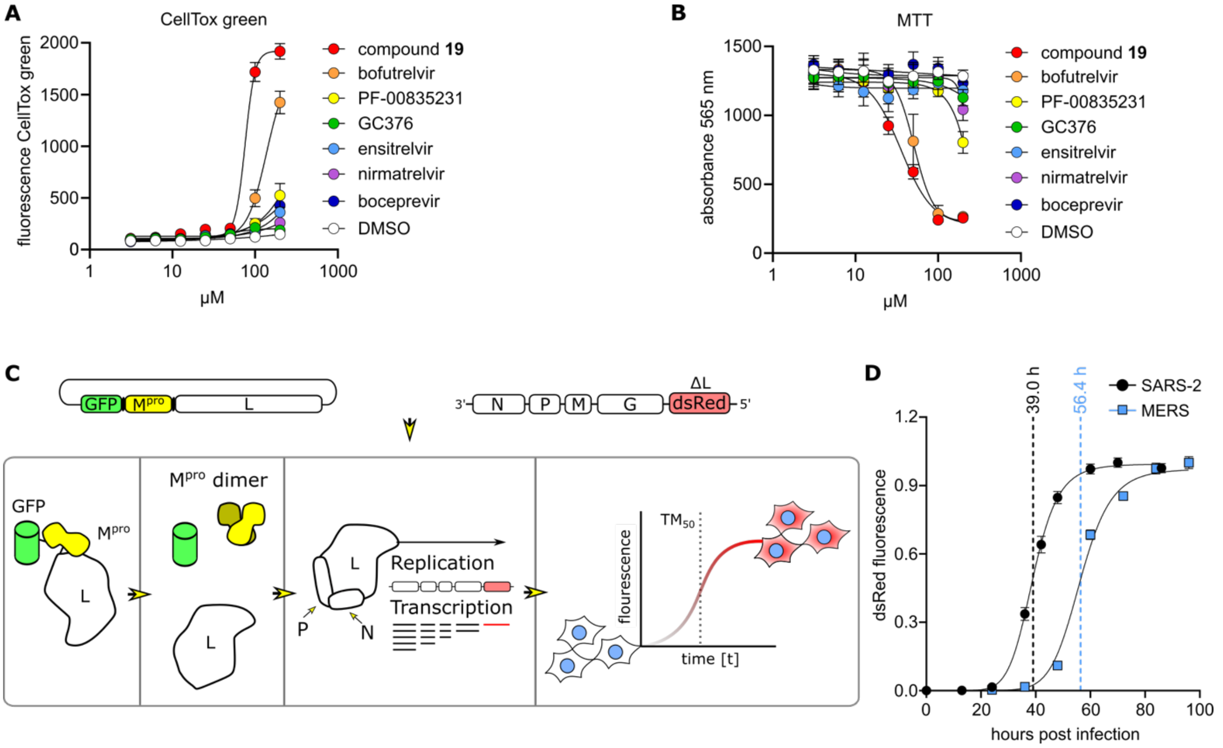

Supplementary Figure 1. Compound cell toxicity and adaptation of the M^{pro}-Off assay for replication kinetics. (**A**) Cell toxicity screening with CellTox green of different compounds tested in non-transfected 293T cells. (**B**) Cell viability screening with MTT of different compounds tested in non-transfected 293T cells. (**C**) The M^{pro}-Off assay was adapted by using GFP-M^{pro}-L and VSV-ΔL-dsRed without applying a protease inhibitor. Fluorescence signals are measured in regular intervals and plotted against time. TM$_{50}$ indicates the time required for the curve to reach half of its maximum value at which the signal plateaus. (**D**) M^{pro}-Off replication kinetics of SARS-CoV-2-M^{pro} wt and MERS-CoV-M^{pro} wt. Data are depicted as means of n = 8 biologically independent replicates per condition. Dotted lines represent the TM$_{50}$ value (hours post infection, hpi). At 80 hpi, both SARS-CoV-2 and MERS-CoV main proteases exhibit a plateauing dsRed signal.

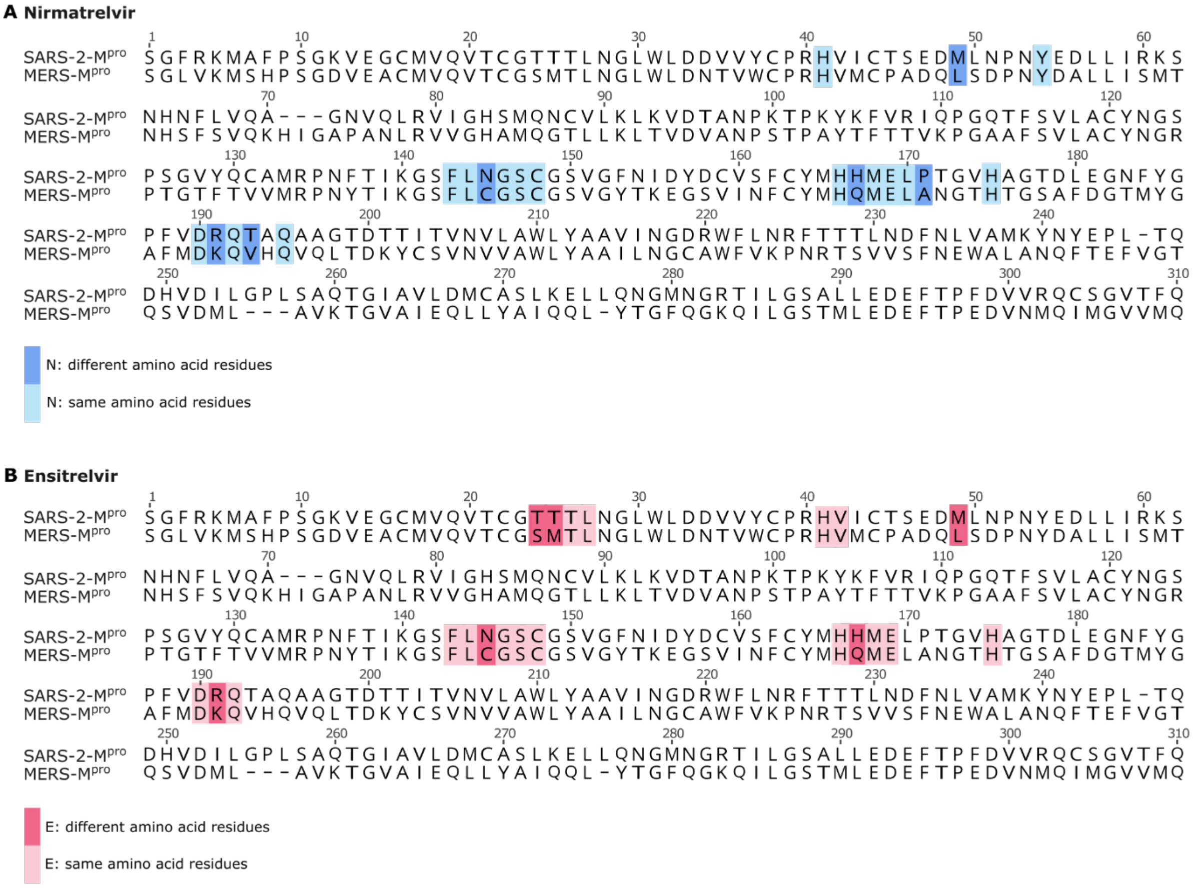

Supplementary Figure 2. Sequence alignments of SARS-CoV-2 and MERS-CoV main proteases highlighting the amino acid residues involved in inhibitor interaction. (**A**) Natural variation of amino acid residues (dark blue) and same residues (light blue) that interact with nirmatrelvir. (**B**) Natural variation in amino acid residues (pink) and same residues (light pink) that interact with ensitrelvir. Interacting residues located within 4 Å of the inhibitor are included.

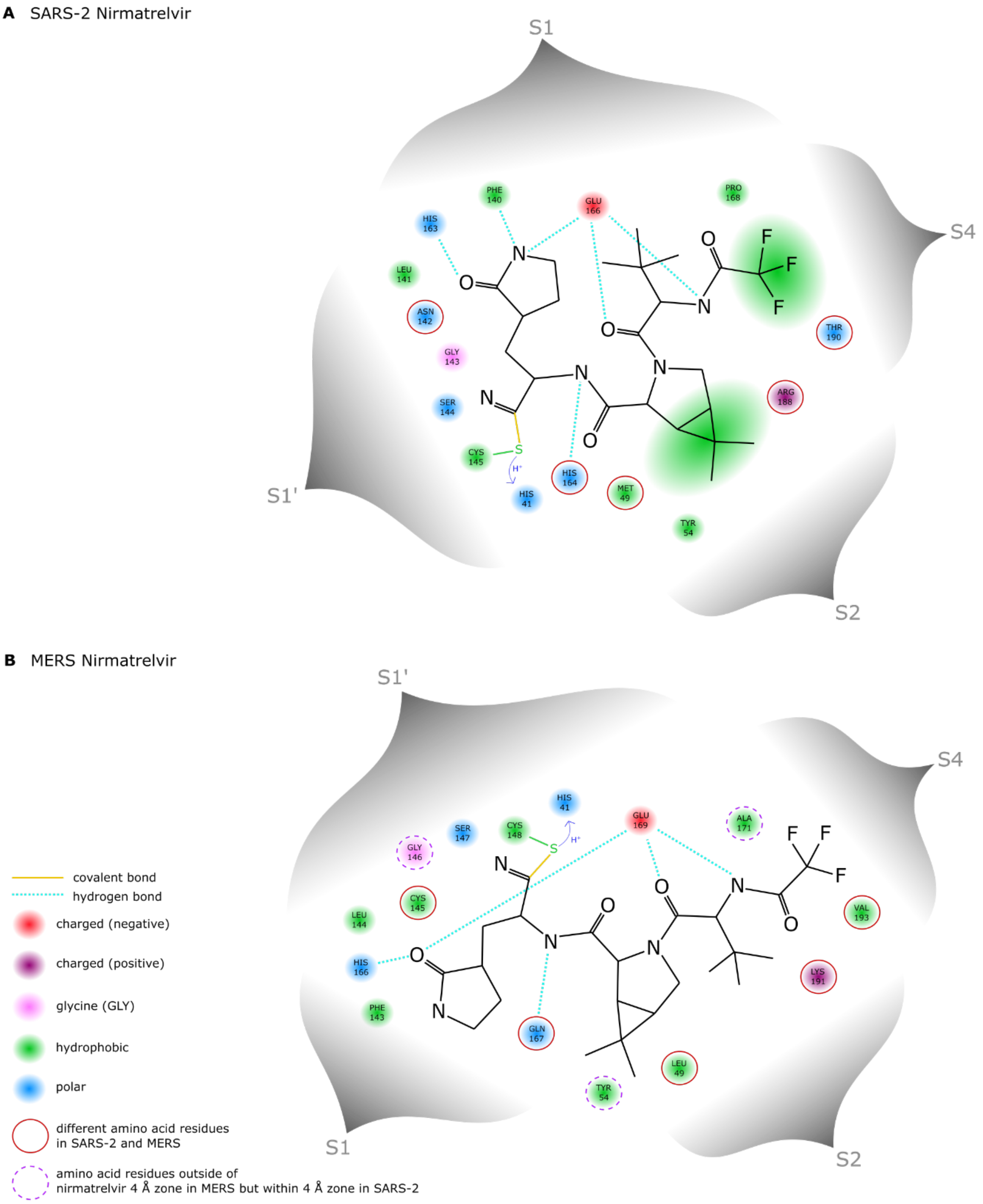

Supplementary Figure 3. Nirmatrelvir 2D binding maps with the catalytic sites of SARS-CoV-2 and MERS-CoV main proteases. (A) The molecular mechanism of nirmatrelvir inhibiting SARS-CoV-2-M^{pro} involves a reversible covalent reaction with the active site cysteine (C145). The nitrile group of nirmatrelvir serves as an electrophilic warhead, forming a covalent bond (yellow line) with the nucleophilic thiolate group of the catalytically active C145, situated within the S1' subsite. Residues 141 - 145 form an oxyanion loop harboring the C145 - H41 catalytic dyad. Histidine (H41) plays a crucial role in deprotonating (blue H^+) the C145 sulfur atom (green S), while G143 and C145 together create an oxyanion hole through their main chain amide NHs. Within the S1 interaction site, nirmatrelvir forms a hydrogen bond (turquoise dotted line) with H163 and three hydrogen bonds with E166. In the S2 subsite, nirmatrelvir's dimethylcyclopropyl proline (DMCP) is surrounded by hydrophobic interactions

(green spheres). Similar interactions exist to stabilize the S4 pocket. (**B**) When examining the MERS-CoV-M^{pro} catalytic site interacting with nirmatrelvir, some amino acid residues (red circles) differ from SARS-CoV-2-M^{pro} interaction. There are the substitutions M49L, H164/167Q and R188/191K. Furthermore, the amino acid stretch 141 - 145, forming an oxyanion loop in SARS-CoV-2-M^{pro}, harbors the substitution N142/145C in MERS-CoV-M^{pro}, potentially reducing polar interactions. The amino acid variation T190/193V exchanges a polar with an apolar residue. G143 in SARS-CoV-2-M^{pro} is essential for building a stabilizing oxyanion hole. The analogous glycine in MERS-CoV-M^{pro} is outside of the nirmatrelvir 4 Å zone (purple dashed circle). The biochemical properties of interacting residues are displayed in red (negatively charged), purple (positively charged), green (hydrophobic) and blue (polar). Different binding types are also listed in the legend. Amino acid abbreviations: ALA (A), ARG (R), ASN (N), CYS (C), GLN (Q), GLU (E), GLY (G), HIS (H), LEU (L), LYS (K), MET (M), PHE (F), PRO (P), SER (S), THR (T), TYR (Y), VAL (V). 2D maps were generated with Inkscape, focusing on the interacting residues located within 4 Å from nirmatrelvir.

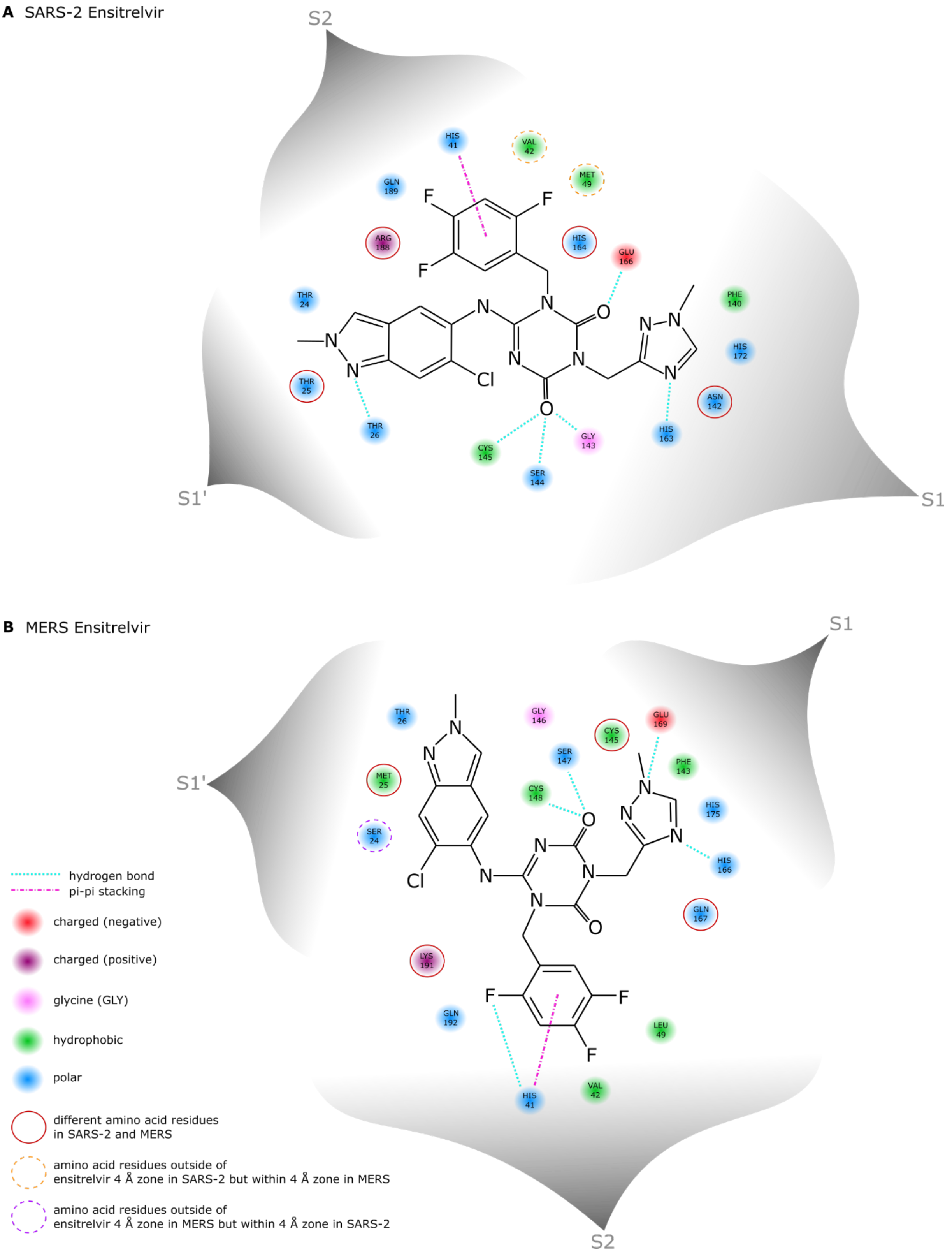

Supplementary Figure 4. Ensitrelvir 2D binding maps with the catalytic sites of SARS-CoV-2 and MERS-CoV main proteases. (A) Ensitrelvir predominantly interacts with the S1', S1 and S2 subsites of SARS-CoV-2-M^{pro}, with fewer interactions within S4. The substrate pocket S1' harbors ensitrelvir's 6-chloro-2-methyl-2H-indazole moiety, which interacts with T26 through a hydrogen bond (turquoise dotted line). The interaction network within the S1 subsite consists mainly of stabilizing hydrogen bonds with E166, F140 and H172. H163 forms a hydrogen bond with the 1-methyl-1H-1,2,4-triazole group of ensitrelvir. In the S2 subsite, the

2,4,5-trifluoromethyl of ensitrelvir engages in a pi-pi stacking interaction (pink dashed line) with the sidechain of H41. C145, G143 and Q189 are involved in the hydrogen bonding network stabilizing ensitrelvir. (**B**) Comparing the catalytic site residues interacting with ensitrelvir in MERS-CoV-M^{pro}, there are natural variations (red circles) from SARS-CoV-2-M^{pro}. In subsite S1', there are the substitutions T24S and T25M. S24 is not within the ensitrelvir 4 Å zone in MERS-CoV-M^{pro} (purple dashed circle). M25 in MERS-CoV-M^{pro} has a hydrophobic side chain, T25 in SARS-CoV-2-M^{pro} a polar side chain. There are the substitutions N142/145C, H164/167Q and R188/191K. M49L is another natural variation, exchanging the hydrophobic amino acid residue of methionine (yellow dashed circle) outside of ensitrelvir's 4 Å zone with a bulkier, positively charged residue of lysine. The biochemical properties of interacting residues are displayed in red (negatively charged), purple (positively charged), green (hydrophobic) and blue (polar). Different binding types are also listed in the legend. Amino acid abbreviations: ARG (R), ASN (N), CYS (C), GLN (Q), GLU (E), GLY (G), HIS (H), LEU (L), LYS (K), MET (M), PHE (F), SER (S), THR (T), VAL (V). 2D maps were generated with Inkscape, focusing on the interacting residues located within 4 Å from ensitrelvir.

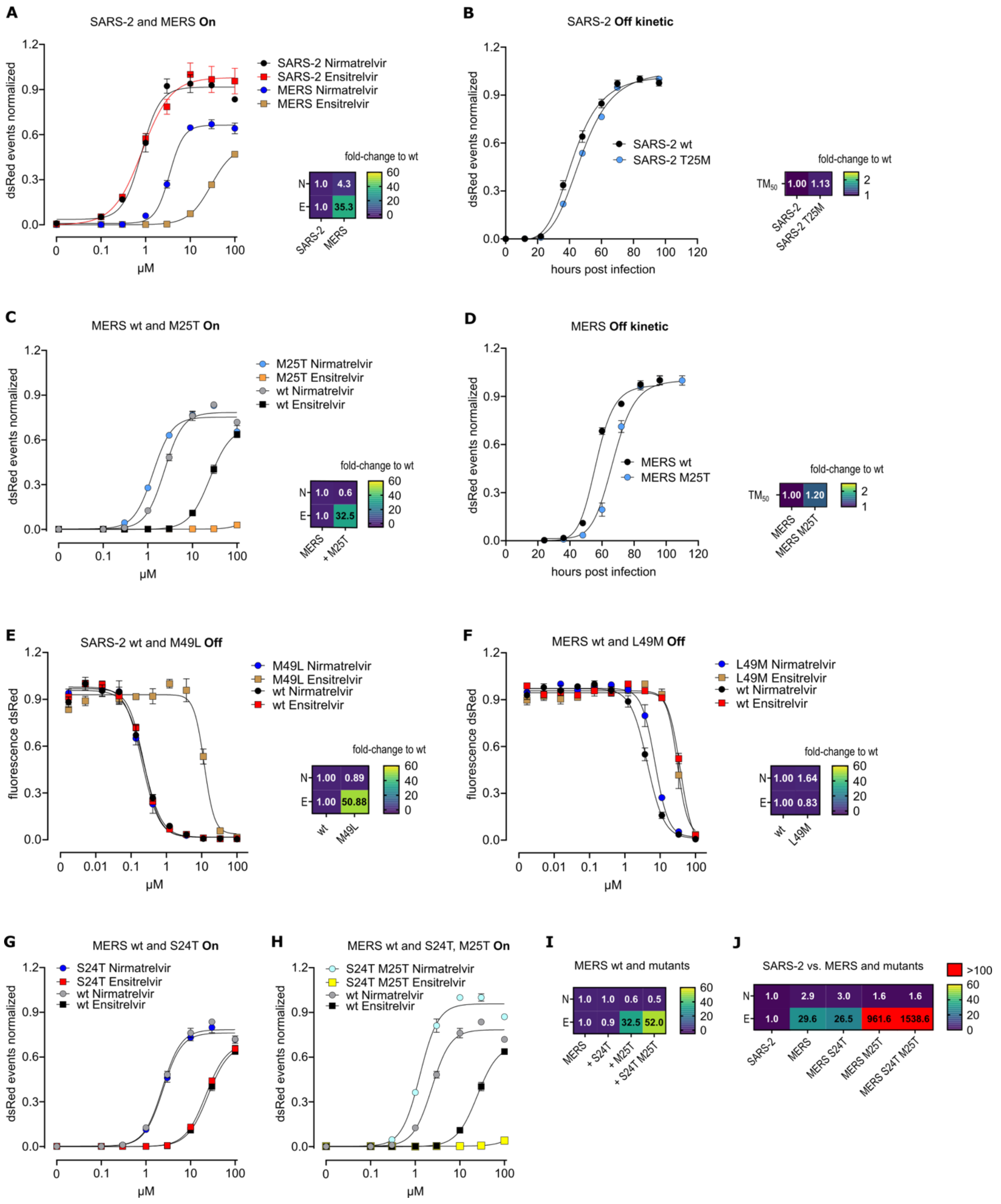

Supplementary Figure 5. Assessing nirmatrelvir (N) and ensitrelvir (E) efficacy against “MERS-CoV to SARS-CoV-2” mutants. (**A**) M^{pro}-On assays of SARS-CoV-2-M^{pro} wt and MERS-CoV-M^{pro} wt with heat map showing IC_{50} fold changes. (**B**) M^{pro}-Off replication kinetics of SARS-CoV-2-M^{pro} wt and mutant T25M with heat map showing TM_{50} fold changes. (**C**) M^{pro}-On assays of MERS-CoV-M^{pro} wt and mutant M25T with heat map showing IC_{50} fold changes. (**D**) M^{pro}-Off replication kinetics of MERS-CoV-M^{pro} wt and mutant M25T with heat map showing TM_{50} fold changes. (**E**) M^{pro}-Off assay of SARS-CoV-2-M^{pro} wt and mutant M49L with heat map showing TM_{50} fold changes. (**F**) M^{pro}-Off assay of MERS-CoV-M^{pro} wt and mutant L49M with heat map showing TM_{50} fold changes. M^{pro}-On assays of MERS-CoV-M^{pro} wt and mutant S24T (**G**) and double mutant S24T / M25T (**H**). (**I**) Heat map showing IC_{50} fold changes of (**G**), (**C**) and (**H**) compared to MERS-CoV-M^{pro} wt. (**J**) Heat map showing IC_{50}

fold changes of SARS-CoV-2-M^{pro} wt compared to MERS-CoV-M^{pro} wt, S24T, M25T or S24T / M25T. M^{pro}-On assays are n = 3 biological replicates per condition. M^{pro}-Off kinetics are n = 8 biological replicates per condition.

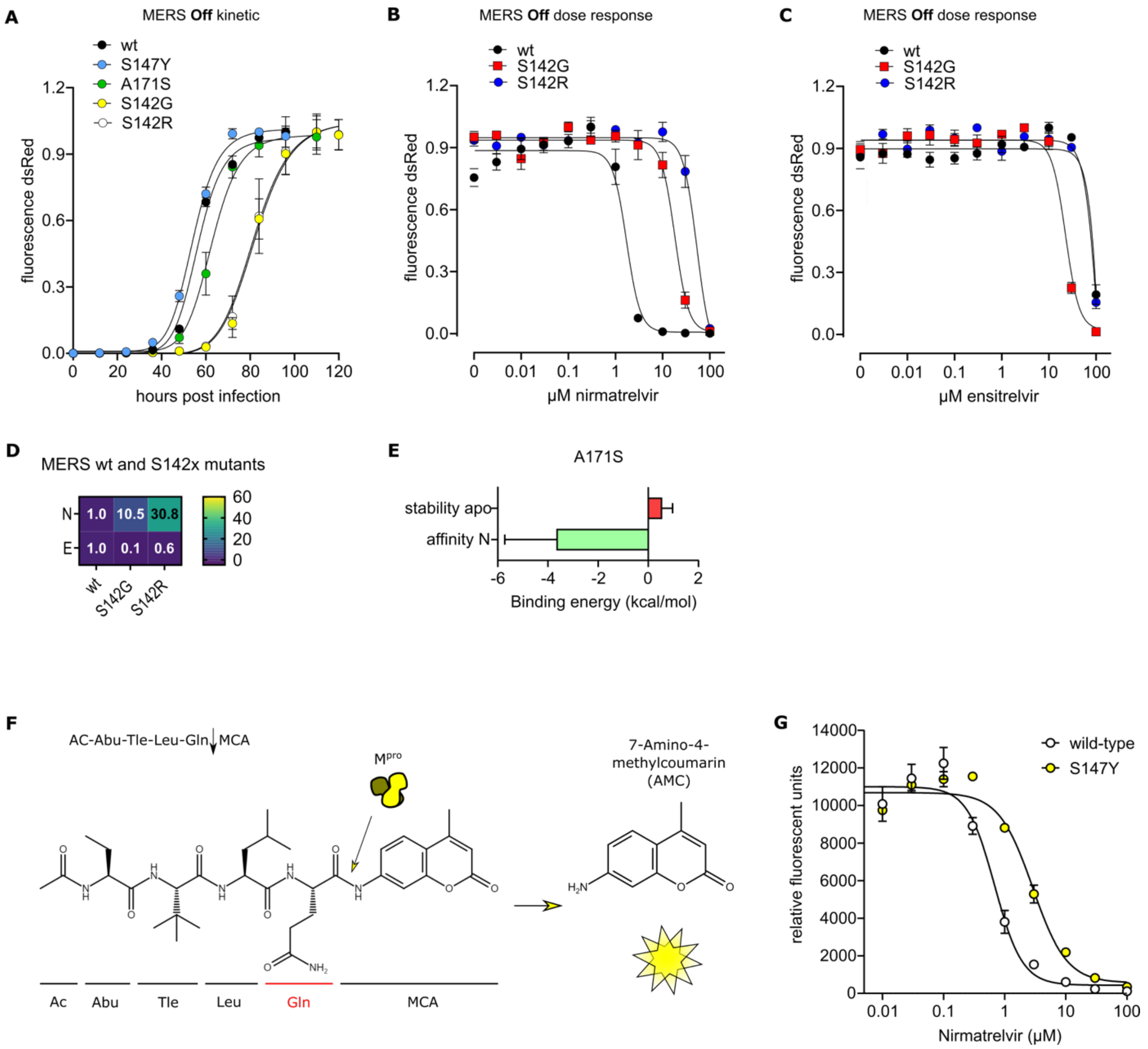

Supplementary Figure 6. Characterization of nirmatrelvir selected MERS-CoV-M^{pro} mutants. (**A**) M^{pro}-Off replication kinetics of MERS-CoV-M^{pro} wt and nirmatrelvir selected MERS-CoV-M^{pro} mutants S147Y, A171S, S142G and S142R. Data are presented as means of n = 8 biological replicates per condition. M^{pro}-Off assays to determine the susceptibility to nirmatrelvir (N) (**B**) and ensitrelvir (E) (**C**) in MERS-CoV-M^{pro} wt and mutants S142G and S142R. Data are presented as means of n = 4 biological replicates per condition. (**D**) Heat map showing IC_{50} fold changes of (**B**) and (**C**). (**E**) Bar chart showing binding energies of A171S, indicating higher nirmatrelvir affinity and mildly reduced apo structure stability. (**F**) Fluorogenic substrate Ac-Abu-Tle-Leu-Gln-MCA cleavage by M^{pro} releases 7-Amino-4-methylcoumarin (AMC). (**G**) Dose response of purified MERS-CoV-M^{pro} proteins wt vs. S147Y mutant.

		7alh	7vh8	8dz0	7zqw	5c3n	7vtc	3tlo	5gwy	6jij	4dcd
**7alh**	apo-SARS2	0.00	0.53	0.72	0.48	0.88	0.74	0.90	0.89	0.79	2.26
**7vh8**	NIR-SARS2	0.53	0.00	0.60	0.39	0.80	0.68	0.97	0.93	0.62	2.38
**8dz0**	ENS-SARS2	0.72	0.60	0.00	0.65	0.75	0.72	0.95	1.01	0.81	2.40
**7zqw**	AG7-SARS1	0.48	0.39	0.65	0.00	0.86	0.75	0.93	0.83	0.74	2.27
**5c3n**	apo-MERS	0.88	0.80	0.75	0.86	0.00	0.67	0.96	1.03	0.84	2.47
**7vtc**	NIR_MERS	0.74	0.74	0.72	0.75	0.67	0.00	0.93	0.87	0.76	2.24
**3tlo**	apo-NL63	0.90	0.97	0.95	0.93	0.96	0.93	0.00	0.55	1.02	2.27
**5gwy**	LEU-NL63	0.89	0.93	1.01	0.83	1.03	0.87	0.55	0.00	0.87	2.34
**6jij**	LEU-MHV	0.79	0.62	0.81	0.74	0.84	0.76	1.02	0.87	0.00	2.24
**4dcd**	DIP-POLIO	2.26	2.38	2.40	2.27	2.47	2.24	2.27	2.34	2.24	0.00

Table 1. Superposition matrix using residues 9 - 194 of chains A (inhibitor binding domain) for M^{pro} structures from different viruses indicated by their PDB ID. Secondary Structure Matching (SSM) superposition on Cα backbone atoms, RMSD in Å. As expected, global atomic RMSD differences between apo and inhibitor-bound forms of the same virus are generally smaller than differences between structures of distant viruses. The differences in inhibitor binding require a detailed local analysis of binding pocket conformation and ligand poses based on experimental structures or virtual docking results.

**VSV-M^{pro} Nirmatrelvir**	**Count in GISAID (3.1.24)**	**Previously described mutation and Pubmed-ID or link)**		**Background of the study / effect of the mutation**
G2D, G2V	7, 15	G2*	37390404	M^{pro} resistance modelling study
F3S	7	F3L	35408866	Review on potential resistance of M^{pro} against protease inhibitors and cleavage sites mutants
F8L	1187			
K12E, K12T	8, 3			
V13A	396			
M17L	12	M17*<br><br>M17I	32853525<br><br>37408369	M^{pro} modelling study, alters dimer stability;<br>Computational mutation study
T21I	21639	T21I	https://www.fda.gov/media/155050/download<br><br>35182772<br><br>36351451<br>36542720	Paxlovid licensing resistance study;<br><br>Frequent variant mutation resistance study;<br><br>SARS-CoV-2 nirmatrelvir resistance studies
C22G, C22T	4,1			
L32I	534			
E47G	298			
Y54C, Y54H, Y54S	13, 20, 16	Y54C<br><br>Y54A<br><br>Y54W	33649700<br>https://www.fda.gov/media/155050/download<br>37390404	In silico M^{pro} screening study;<br><br>Paxlovid licensing resistance study;<br><br>M^{pro} resistance modelling study
L57F	26			
Q74L	18			
H80P	10			
T93P	11			
T98I, T98S	29, 83	T98I	doi: 10.1093/ofid/ofad500.431	Clinical Paxlovid treatment study
P99L	193	P99L	32853525<br>36870228<br>37408369	M^{pro} modelling study;<br>Modelled increased dimer stability;<br>Computational mutation study
K100N	2474			
T111N	39			
Y118H	17			
N119D	210			
G124D	125			
Y126S, Y126F	22, 145			
C128Y	12	C128*	32853525	M^{pro} modelling study,<br>alters dimer stability
A129T, A129S	324, 251	A129V	32853525	M^{pro} modelling study
G138S	25			

S139P	11	S139P	37390404<br>36351451	M^{pro} resistance modelling study;<br>SARS-CoV-2 nirmatrelvir resistance study
L141F	41	L141*	37390404	M^{pro} resistance modelling study
S144A	21	S144A	https://www.fda.gov/media/155050/download<br>37637734<br>36351451	Paxlovid licensing resistance study;<br><br>SARS-CoV-2 in vitro resistance studies
D153N	38			
L167F	29	L167F	https://www.fda.gov/media/155050/download<br><br>35731933<br><br>36351451<br>36625640	Paxlovid licensing resistance study;<br><br>Evolutionary study of anti-nirmatrelvir mutations;<br><br>SARS-CoV-2 in vitro resistance studies
P168S	547	P168S<br>P168S<br>P168R<br>P168R	35731933<br>36989354<br>36029764<br>37390404	Evolutionary study of anti-nirmatrelvir mutations;<br>In vitro resistance study;<br>Mutant enzyme activity study;<br>M^{pro} resistance modelling study
F181S	15			
P184S	4993	P184S<br>P184L/S	32853525<br>37408369	M^{pro} modelling study;<br>Computational mutation study
R188W	8	R188G	https://www.fda.gov/media/155050/download<br>35731933	Paxlovid licensing resistance study;<br>Evolutionary study of anti-nirmatrelvir mutations
Q192R	54	Q192*	https://www.fda.gov/media/155050/download<br>37637734	Paxlovid licensing resistance study;<br><br>SARS-CoV-2 in vitro resistance study
A194S	1004			
D197Y, D197A	45, 4	D197*	36590977	In-silico evolutionary study of SARS-CoV-2
T198I	2272	T198I	doi: 10.1093/ofid/ofac492.972<br>32853525	Clinical Xocova treatment study<br>M^{pro} modelling study
I200V, I200T	82, 17			
V202F	38			
N203H, N203K, N203S, N203D	11, 37, 49, 79			
V204F	10			
A206T	6			
W207C, W207R	25, 17	W207L	doi: 10.1093/ofid/ofad500.431	Clinical Paxlovid treatment study

L208W	5			
A210D, A210S, A210T	59, 729, 27			
A211V	8			
D216Y, D216A	122, 4			
F219S	16	F219L	22345451	Compensates temperature sensitivity in MHV M^{pro} (Omicron M^{pro} is also temp. sens.)
L220P	12	L220F<br>L220P	37408369<br>32931703	Computational mutation study;<br>M^{pro} modelling study
R222L	742			
D229G	7			
L232R	6	L232R	32853525	M^{pro} modelling study
A234D, A234T	31, 1024	A234S<br><br>A234V	doi: 10.1093/ofid/ofac492.972<br>32853525<br>37408369<br>32931703	Clinical Xocova treatment study;<br><br>M^{pro} modelling study;<br><br>Computational mutation study
T257N	25			
A260D	84	A260V	32853525<br>37408369<br>32931703	M^{pro} modelling study;<br>Computational mutation study;<br>M^{pro} modelling study;
A266T	531	A266V	32853525<br>37408369<br>32931703	M^{pro} modelling study;<br>Computational mutation study;<br>M^{pro} modelling study;
L268F	13	L268N	36029764	Mutant enzyme activity study
M276R	8			
N277K	287			
I281R	2			
L282S	18			
D289T	4			
T292K	3	T292M<br>T292A	36029764<br>37254750	Mutant enzyme activity studies
D295G, D295N, D295V, D295Y	6, 5, 2, 8	D295G<br>D295N	36029764<br>37254750	Mutant enzyme activity studies
V296F, V296G	13, 3			
R298G	280			
Q299K, Q299P, Q299R	10, 1, 35	Q299A<br>Q299H	36029764<br>37390404	Mutant enzyme activity study;<br>M^{pro} resistance modelling study
C300R	8		37390404	M^{pro} resistance modelling study
F305L	130	F305L	35408866	Review on potential resistance of M^{pro} against protease inhibitors and cleavage sites mutants
**VSV-M^{pro} Ensitrelvir**	**Frequency in GISAID**		**Previously described**	
S1R		S1H<br>S1N	37390404<br>35408866	M^{pro} resistance modelling study;

				Review on potential resistance of M^{pro} against protease inhibitors and cleavage sites mutants
G2V	15,	G2*	37390404	M^{pro} resistance modelling study
K12E, K12T	8, 3			
V18A	291			
C22F, C22Y	10, 6			
T25N, T25A	18, 224	T25C<br>T25E/H<br>T25A	36029764<br>37390404<br>37254750	Mutant enzyme activity study;<br>M^{pro} resistance modelling study;<br>Mutant enzyme activity study
D33V	21			
C38F	30			
R40K	16			
V42L	459			
C44Y, C44R, C44G	20, 27, 10	C44G	37390404	M^{pro} resistance modelling study
E47A	266			
D48Y	126		36989354	In-vitro resistance study
M49L	338	M49L<br>M49L/I<br>M49L	37454219<br>36989354<br>37408369	SARS-CoV-2 resistance and transmission study;<br>In-vitro resistance study;<br>Computational mutation study
P52H, P52L	20, 40	P52L	37390404	M^{pro} resistance modelling study
D56Y	19			
L57F	26			
F66V	515	F66W	36029764	Mutant enzyme activity study
Q69P	7	Q69C	36029764	Mutant enzyme activity study
I78M	46			
H80P, H80R	10, 33			
M82L	104			
Q83R	158			
C85S, C85G	53, 10			
L89F	173242	L89F	35182772<br>32853525<br>37408369	Frequent variant mutation resistance study;<br>M^{pro} modelling study;<br>Computational mutation study
K102N	83			
R105C	61	R105H	32853525	M^{pro} modelling study
G109R	212			
L115F	5			
A116T	513	A116V<br>A116*	32853525<br>37390404	M^{pro} modelling study;<br><br>M^{pro} resistance modelling study
C117G	3			
P122S, P122L	254, 14			
V125A, V125G	8, 4			
Q127R	15			
C128Y	12			
A129T	324	A129V	32853525	M^{pro} modelling study

R131K	21			
L141F, L141I	41, 27	L141*	37390404	M^{pro} resistance modelling study
N142D	229	N142L	36326185	Computational and in-vitro nirmatrelvir resistance study
H163Q	6		38070810	In vitro resistance study
H164N	4853	H164N	https://www.fda.gov/media/155050/download	Paxlovid licensing resistance study
E166K, E166A	16, 11	E166A<br>E166M<br>E166A	36625640<br>36326185<br>37390404	SARS-CoV-2 nirmatrelvir resistance studies<br>Computational and in-vitro nirmatrelvir resistance study;<br>M^{pro} resistance modelling study
L167F	29	L167F	https://www.fda.gov/media/155050/download<br><br>35731933<br><br>36351451<br>36625640	Paxlovid licensing resistance study;<br><br>Evolutionary study of anti-nirmatrelvir mutations;<br><br>SARS-CoV-2 nirmatrelvir resistance studies
P184H	4385	P184L/S	32853525	M^{pro} modelling study
R188W	8	R188G	https://www.fda.gov/media/155050/download	Paxlovid licensing resistance study
Q189P, Q189L, Q189H	31, 45, 42	Q189E/I<br>Q189*	36326185<br>35731933	Computational and in-vitro nirmatrelvir resistance study;<br>Evolutionary study of anti-nirmatrelvir mutations
Q192P, Q192R	17, 54	Q192T<br>Q192P/*	36326185<br>37637734	Computational and in-vitro nirmatrelvir resistance study;<br>SARS-CoV-2 in vitro resistance study
T198P	33	T198P	32853525	M^{pro} modelling study
N203K, N203D, N203S	37, 79, 49			
V204F	10			
A206T	6			
W207R	17	W207L	doi: 10.1093/ofid/ofad500.431	Clinical Paxlovid treatment study
A210T, A210V	27, 34			
D216G	16			
F219L, F219S	30, 16	F219L	22345451	Compensates temperature sensitivity in MHV M^{pro} (Omicron M^{pro} is also temp. sens.)
N221K	40	N221W	36029764	Mutant enzyme activity study
N228S	683			
E240K	7			
L242I	134			

T243I	2916			
D245Y	26			
H246R	193	H246Y	doi: 10.1093/ofid/ofac492.972	Clinical Xocova treatment study
L250P	11			
Q256R	35			
G258R	29	G258Q	(36029764	Mutant enzyme activity study
A260T	455	 A260V	32853525<br>37408369	M^{pro} modelling study;<br>Computational mutation study
C265Y	5			
M276V, M276L, M276R	1240, 362, 8			
G278V, G278E	32, 299			
R279C, R279H	4919, 1396	R279C<br>R279C<br>R279C	32853525<br>36870228<br>32931703	M^{pro} modelling study;<br>Modelled decreased enzyme stability;<br>Modelling study of SARS-CoV-2 mutants
L287S	10			
D289E	10			
E290G	5			
T292I, T292P	20	T292M	36029764	Mutant enzyme activity study
D295A	2	D295G	36029764	Mutant enzyme activity study
R298G	280			
Q299P, Q299K	1, 10	Q299A	36029764	Mutant enzyme activity study
C300R, C300Y	8, 5		34399606	C300 glutathionylation paper
G302V	8	G302C/S	35408866	Review on potential resistance of M^{pro} against protease inhibitors and cleavage sites mutants
F305L	130	F305L	35408866	Review on potential resistance of M^{pro} against protease inhibitors and cleavage sites mutants

**VSV-Spike-M^{pro} Nirmatrelvir**	**Frequency in GISAID**		**Previously described**	
D34N	317			
V35A	143			
V42G	-			
E47G	298			
R105L	594	R105H	32853525	M^{pro} modelling study
Q110K	9			
E166K, E166V	16, 50	E166V<br>E166V<br>E166V<br>E166V<br>E166A	doi: 10.1093/ofid/ofad500.431<br>36029764<br>36351451<br>36542720<br>37390404	Clinical Paxlovid treatment study;<br>SARS-CoV-2 nirmatrelvir resistance studies;<br>M^{pro} resistance modelling study

P168Q	20	P168S<br>P168S<br>P168S<br>P168R	35731933<br>36989354<br>36029764<br>37390404	In vitro resistance studies;<br>Mutant enzyme activity study;<br>M^{pro} resistance modelling study
E178A	69			
V247G	1260			
D248A	31	D248E	37408369	Computational mutation study
G278R	692			
F305L	130	F305L	35408866	Review on potential resistance of M^{pro} against protease inhibitors and cleavage sites mutants
**VSV-Spike-M^{pro} Ensitrelvir**	**Frequency in GISAID**		**Previously described**	
M6T	35	M6I	36351451	SARS-CoV-2 nirmatrelvir resistance study
V35A	143	V35L	32931703	M^{pro} modelling study
E166K	16	see above	see above	see above

Table 2. Overview of all substitutions generated during selection experiments with chimeric VSV-M^{pro} variants. **GISAID**: Global Initiative on Sharing All Influenza Data, reference data bank with most SARS-CoV-2 sequence depositions.

Position nct	Position aa	Codon wt	Codon mut	substitution	% snp	Localization
8	3	TTG	TCG	L3S	48.4%	cleavage site
17	6	ATG	AAG	M6K	6.0%	cleavage site
31	11	GGT	AGT	G11S	22.3%	allosteric
71	24	AGC	ATC	S24I	8.1%	near catalytic site
241	81	GTG	ATG	V81M	5.4%	allosteric
335	112	GGC	GAC	G112D	10.2%	allosteric
362	121	TAC	TTC	Y121F	60.2%	near catalytic site
634	121	TAC	AAC	Y121N	5.9%	near catalytic site
401	134	CGA	CAA	R134Q	96.9%	allosteric
403	135	CCG	ACG	P135T	86.5%	allosteric
418	140	AAG	GAG	K140E	9.5%	near catalytic site
418	140	AAG	GAG	K140E	7.1%	near catalytic site
424	142	AGC	GGC	S142G	89.0%	catalytic site
424	142	AGC	GGC	S142G	78.0%	catalytic site
424	142	AGC	GGC	S142G	97.0%	catalytic site
424	142	AGC	CGC	S142R	33.0%	catalytic site
440	147	TCC	TAC	S147Y	13.4%	catalytic site
464	155	AAG	ACG	K155T	11.1%	allosteric
480	160	ATT	ATG	I160M	15.3%	allosteric
511	171	GCG	TCG	A171S	31.6%	catalytic site
556	186	GGT	TGT	G186C	7.0%	near catalytic site
556	186	GGT	AGT	G186S	8.1%	near catalytic site
557	186	GGT	GTT	G186V	7.8%	near catalytic site
564	188	TTT	TTG	F188L	27.2%	catalytic site
599	200	GAC	GGC	D200G	13.0%	near catalytic site
598	200	GAC	TAC	D200Y	28.9%	near catalytic site
607	203	TGT	CGT	C203R	84.4%	allosteric
611	204	TCT	TTT	S204F	29.2%	allosteric
610	204	TCT	CCT	S204P	37.6%	allosteric
616	206	AAT	GAT	N206D	95.3%	allosteric
616	206	AAT	GAT	N206D	98.2%	allosteric
616	206	AAT	GAT	N206D	22.5%	allosteric
616	206	AAT	GAT	N206D	23.7%	allosteric
616	206	AAT	GAT	N206D	98.4%	allosteric
616	206	AAT	GAT	N206D	86.1%	allosteric
616	206	AAT	GAT	N206D	24.6%	allosteric
616	206	AAT	GAT	N206D	78.7%	allosteric
616	206	AAT	GAT	N206D	6.6%	allosteric
616	206	AAT	CAT	N206H	8.1%	allosteric
618	206	AAT	AAG	N206K	88.6%	allosteric
618	206	AAT	AAA	N206K	14.2%	allosteric
618	206	AAT	AAG	N206K	20.0%	allosteric
618	206	AAT	AAG	N206K	80.4%	allosteric
618	206	AAT	AAG	N206K	9.1%	allosteric
617	206	AAT	AGT	N206S	7.6%	allosteric

617	206	AAT	AGT	N206S	14.2%	allosteric
617	206	AAT	ACT	N206T	94.9%	allosteric
617	206	AAT	ACT	N206T	45.7%	allosteric
617	206	AAT	ACT	N206T	22.3%	allosteric
617	206	AAT	ACT	N206T	77.2%	allosteric
622	208	GTC	TTC	V208F	64.9%	allosteric
622	208	GTC	TTC	V208L	5.9%	allosteric
626	209	GCC	GAC	A209D	8.0%	allosteric
625	209	GCC	ACC	A209T	78.4%	allosteric
625	209	GCC	ACC	A209T	32.5%	allosteric
625	209	GCC	ACC	A209T	6.7%	allosteric
625	209	GCC	ACC	A209T	24.4%	allosteric
625	209	GCC	ACC	A209T	11.4%	allosteric
625	209	GCC	ACC	A209T	10.0%	allosteric
628	210	TGG	CGG	W210R	92.2%	allosteric
632	211	CTC	CCC	L211P	7.7%	allosteric
634	212	TAC	GAC	Y212D	96.3%	allosteric
634	212	TAC	CAC	Y212H	19.5%	allosteric
637	213	GCC	ACC	A213T	27.6%	allosteric
641	214	GCG	GAG	A214E	50.7%	allosteric
668	223	GTA	GGA	V223G	12.4%	allosteric
683	228	ACT	AAT	T228N	75.8%	allosteric
698	233	TTC	TGC	F233C	57.0%	allosteric
699	233	TTC	TTA	F233L	91.6%	allosteric
697	233	TTC	CTC	F233L	44.8%	allosteric
698	233	TTC	TCC	F233S	93.9%	allosteric
698	233	TTC	TCC	F233S	79.8%	allosteric
697	233	TTC	GTC	F233V	4.6%	allosteric
697	233	TTC	GTC	F233V	15.0%	allosteric
700	234	AAT	TAT	N234Y	9.1%	allosteric
700	234	AAT	TAT	N234Y	93.2%	allosteric
706	236	TGG	GGG	W236G	38.1%	allosteric
706	236	TGG	CGG	W236R	6.6%	allosteric
709	237	GCC	ACC	A237T	8.1%	allosteric
718	240	AAC	GAC	N240D	97.5%	allosteric
718	240	AAC	GAC	N240D	97.0%	allosteric
734	245	TTT	TCT	F245S	9.7%	allosteric
734	245	TTT	TCT	F245S	15.7%	allosteric
734	245	TTT	TCT	F245S	95.7%	allosteric
734	245	TTT	TCT	F245S	14.9%	allosteric
772	258	ACA	CCA	T258P	9.3%	allosteric
775	259	GGG	TGG	G259W	10.6%	allosteric
775	259	GGG	TGG	G259W	94.0%	allosteric
778	260	GTT	TTT	V260F	96.4%	allosteric
779	260	GTT	GGT	V260G	62.9%	allosteric
779	260	GTT	GGT	V260G	10.6%	allosteric

788	263	GAG	GGG	E263G	7.1%	allosteric
787	263	GAG	AAG	E263K	3.7%	allosteric
787	263	GAG	AAG	E263K	16.4%	allosteric
787	263	GAG	AAG	E263K	5.8%	allosteric
787	263	GAG	AAG	E263K	14.3%	allosteric
787	263	GAG	AAG	E263K	76.4%	allosteric
797	266	CTT	CCT	L266P	92.6%	allosteric
797	266	CTT	CCT	L266P	64.5%	allosteric
803	268	GCC	CCC	A268P	23.2%	allosteric
806	269	ATA	AGA	I269R	49.1%	allosteric
863	288	GAA	GGA	E288G	90.3%	allosteric
866	289	GAT	GGT	D289G	17.7%	allosteric
866	289	GAT	GGT	D289G	71.9%	allosteric
866	289	GAT	GGT	D289G	9.7%	allosteric
866	289	GAT	GGT	D289G	90.3%	allosteric
865	289	GAT	AAT	D289N	7.2%	allosteric
865	289	GAT	AAT	D289N	20.4%	allosteric
865	289	GAT	TAT	D289Y	6.0%	allosteric
865	289	GAT	TAT	D289Y	88.2%	allosteric
865	289	GAT	TAT	D289Y	83.2%	allosteric
875	292	ACG	AAG	T292K	18.7%	allosteric
878	293	CCG	CAG	P293Q	7.6%	allosteric
880	294	GAG	AAG	E294K	7.5%	allosteric
887	296	GTA	GCA	V296A	10.0%	allosteric
887	296	GTA	GCA	V296A	16.5%	allosteric
887	296	GTA	GGA	V296G	68.3%	allosteric
887	296	GTA	GGA	V296G	87.8%	allosteric
887	296	GTA	GGT	V296G	6.5%	allosteric
887	296	GTA	GGA	V296G	39.1%	allosteric
887	296	GTA	GGA	V296G	85.1%	allosteric
886	296	GTA	TTA	V296L	95.5%	allosteric
886	296	GTA	TTA	V296L	11.0%	allosteric
896	299	CAG	CCG	Q299P	57.9%	allosteric
899	300	ATA	AAA	I300K	97.7%	cleavage site
899	300	ATA	AAA	I300K	97.7%	cleavage site

Table 3. Mutations selected with recombinant VSV-MERS-M^{pro}. Columns: position nucleotide (nct), position amino acid (aa), codon wild-type (wt), codon mutant (mut), amino acid substitution, percentage of the mutation / single nucleotide polymorphism within each well (% snp), localization of the substitution. Position numbers relate to MERS-CoV-M^{pro} (**Sequence 3**, NCBI: KF600615.1).

Name	Sequence (5'-3' direction)
**VSV-G-M^{pro}-L**	
G-33n-before-KpnI-for	GAACCGGTCCTGCTTTCACC
G-rev	CTTTCCAAGTCGGTTCATCTC
G-cut1-7aa-for	GAGATGAACCGACTTGGAAAGATCACTAGCGGTGTATTGCAG
cut2-7aa-L-rev	GTCTCAAAATCGTGGACTTCCATTGTTACTTTTCTTACACCGGACTG
G-cut1-8aa-for	GAGATGAACCGACTTGGAAAGTCAATCACTAGCGGTGTATTGCAG
cut2-8aa-L-rev	GTCTCAAAATCGTGGACTTCCATGTATGTTACTTTTCTTACACCGGACTG
L-for	ATGGAAGTCCACGATTTTGAGACCGACG
L-33n-after-HpaI-rev	ATGGAAGTCCACGATTTTGAGACCGACG
**MERS-M^{pro}-On-Nt-QtoN**	
Hygro-P-for	CTGTTTTGACCTCCATAGAAGATTCTAGAGCTAGCATGGATAATCTCACAAAAGTTC
P-GGSG-rev	GCTCCCTCCGCCGCTTCCGCCATCTGATACTGCTTCTGATTGG
MERS-On-N-term-QtoN-for	GGCGGAAGCGGCGGAGGGAGCGGGGGCGGGAGCGGATCAATCACTAGCGGTGTATTGAACAGTGGTTTGGTC
MERS-On-C-term-rev	GCCGGATCCACCGCCTGAGCCGCCTCCGGACCCTCCGTATGTTACTTTTCTTACACCGGAC
GGSG-P-for	GGCTCAGGCGGTGGATCCGGCGTTTGGTCTCTCTCAAAGACAT
Hygro-P-rev	GAGGGAGAGGGGCGGATCCCCTTAATTAACTACAGAGAATATTTGACTCTCGC
**MERS-M^{pro}-Off**	
Blasti-L-for	CTGTTTTGACCTCCATAGAAGATTCTAGAGCTAGCATGGAAGTCCACGATTTTGAG
L-blasti-rev	GAGGGAGAGGGGCGGATCCCCTTAATTAATTAATCTCTCCAAGAGTTTTCCTC
Blasti-for	CATTCGATTAGTGAACGGATCTC
GFP-rev	CTTGTACAGCTCGTCCATGCC
GFP-cut1-MERS-Off-7aa-for	GGCATGGACGAGCTGTACAAGATCACTAGCGGTGTATTGCAG
7aa-MERS-Off-cut2-L-rev	GTCTCAAAATCGTGGACTTCCATTGTTACTTTTCTTACACCGGACTG
L-for	ATGGAAGTCCACGATTTTGAGACCGACG
L-33n-after-HpaI-rev	GATGTTGGGATGGGATTGGC
**Mutation primers**	
SARS-2-T25A-for	CAAGTAACTTGTGGTACAGCTACAC
SARS-2-T25A-rev	GACCGTTAAGTGTAGCTGTACC
SARS-2-T25N-for	CAAGTAACTTGTGGTACAAATACACTTAACG
SARS-2-T25N-rev	GCCAAAGACCGTTAAGTGTATTTGTAC
SARS-2-T25M-for	CAAGTAACTTGTGGTACAATGACACTTAAC
SARS-2-T25M-rev	GACCGTTAAGTGTCATTGTACCAC
SARS-2-M49L-for	CACCTCTGAAGACCTGCTTAACC
SARS-2-M49L-rev	CATAATTAGGGTTAAGCAGGTCTTCAG

MERS-M25T-for	CCTGTGGTAGCACTACACTTAATG
MERS-M25T-rev	CCAGAGTCCATTAAGTGTAGTGCTAC
MERS-S142G-for	CGAATTATACGATTAAGGGTGGCTTTTTG
MERS-S142G-rev	CCACACAAAAAGCCACCC
MERS-S142R-for	CGAATTATACGATTAAGGGTCGCTTTTTG
MERS-S142R-rev	CCACACAAAAAGCGACCC
MERS-S147Y-for	GTAGCTTTTTGTGTGGTTACTGTGG
MERS-S147Y-rev	CGACAGAACCACAGTAACCAC
MERS-A171S-for	CACCAAATGGAACTCTCGAACG
MERS-A171S-rev	GGTATGAGTACCGTTCGAGAGTTC
MERS-S24T-for	CAAGTAACCTGTGGTACAATGACAC
MERS-S24T-rev	GAGTCCATTAAGTGTCATTGTACCAC
MERS-S24T-M25T-for	CCAAGTAACCTGTGGTACAACTACAC
MERS-S24T-M25T-rev	CCAGAGTCCATTAAGTGTAGTTGTACC
MERS-L49M-for	CCGGCGGACCAGATGTCTGACC
MERS-L49M-rev	GCGTCATAGTTTGGGTCAGACATCTGGTC
**Other On constructs**	
SARS-2-On-N-term-QtoN-for	CACCTCAGCTGTTTTGAACAGTGG
SARS-2-On-N-term-QtoN-rev	CTAAAACCACTGTTCAAAACAGC
SARS-1-On-N-term-QtoN-for	GGCGGAAGCGGCGGAGGGAGCGGGGGCGGGAGCGGAAGTA<br>TCACGTCTGCTGTGCTCAACTCAGGCTTCAG
SARS-1-GGSG-On-rev	GCCGGATCCACCGCCTGAGCCGCCTCCGGACCCTCCTTTGAC<br>TATTTTTTTGAACTTACCTTG
HKU9-On-N-term-QtoN-for	GGCGGAAGCGGCGGAGGGAGCGGGGGCGGGAGCGGAAGCG<br>TCGCCAGTGCTGCGCTCAACGCGGGTCTTACTC
HKU9-GGSG-On-rev	GCCGGATCCACCGCCTGAGCCGCCTCCGGACCCTCCTCGAAA<br>CATAGATTGAAATTTACCTTG
HCoV-NL63-On-N-term-QtoN-for	GGCGGAAGCGGCGGAGGGAGCGGGGGCGGGAGCGGAATCA<br>GTTACAATAGTACCTTGAACAGCGGACTG
HCoV-NL63-GGSG-On-rev	GCCGGATCCACCGCCTGAGCCGCCTCCGGACCCTCCAAGCCC<br>GAATATAACCTTTCCTG
HCoV-229E-On-N-term-QtoN-for	GGCGGAAGCGGCGGAGGGAGCGGGGGCGGGAGCGGAGTAT<br>CTTATGGCTCAACGCTCAACGCCGGCTTGCGC
HCoV-229E-GGSG-On-rev	GCCGGATCCACCGCCTGAGCCGCCTCCGGACCCTCCAAACAT<br>GGATGTAGTCTTACCAGATTG
MHV-A59-On-N-term-QtoN-for	GGCGGAAGCGGCGGAGGGAGCGGGGGCGGGAGCGGATCAG<br>TCACCACTTCATTTCTCAACTCCGGGATAG
MHV-A59-GGSG-On-rev	GCCGGATCCACCGCCTGAGCCGCCTCCGGACCCTCCTTTTAT<br>TACTCTAGTCCTTTTACTCTGCAG
Polio-On-N-term-QtoN-for	GGCGGAAGCGGCGGAGGGAGCGGGGGCGGGAGCGGAACCA<br>TTCGGACAGCAAAGGTAAACGGACCAGGGTTC
Polio-GGSG-On-rev	GCCGGATCCACCGCCTGAGCCGCCTCCGGACCCTCCAGGTCT<br>CATCCACTGGATTTC
***E.coli* M^{pro} expression plasmid**	
M^{pro}-expr.-S147Y-for	GCTTTCTGTGTGGTTACTGCGG
M^{pro}-expr.-S147Y-rev	CGCTACCGCAGTAACCACAC

Table 4. Cloning oligonucleotides / primers.

Alignments 1 to 7

Alignment 1: Amino acid sequence alignments of SARS-CoV-2 and -1 main proteases in fasta format.

Alignment 2: Amino acid sequence alignments of SARS-CoV-2 and MERS-CoV main proteases in fasta format.

Alignment 3: Amino acid sequence alignments of SARS-CoV-2 and MERS-CoV spike in fasta format.

Alignment 4: Amino acid sequence alignments of SARS-CoV-2 and MERS-CoV spike in fasta format.

Alignment 5: Amino acid main protease sequence alignments of 494 samples from different MERS-CoV clades in fasta format.

Alignment 6: Amino acid sequence alignments of main protease representatives from MERS-CoV clades A-C with outgroup main proteases in fasta format.

Alignment 7: Amino acid sequence alignments of SARS-CoV-2 and MERS-CoV spike in fasta format.

Plasmids 1 to 7

Plasmid 1: VSV-Indiana antigenome pBluescript expression plasmid under a T7 polymerase promoter and terminator with hepatitis-D ribozyme and ampicillin resistance.

Plasmid 2: VSV-Indiana plasmid with insertion of the MERS-CoV M^{pro} and cognate cleavage sites replacing the VSV intergenic region between VSV-G and VSV-L.

Plasmid 3: Lentiviral expression vector modified from Addgene pLenti CMVie-IRES-BlastR accession #119863 by replacing blasticidin resistance with hygromycin resistance to generate pLenti CMVie-IRES-HygroR.

Plasmid 4: pLenti CMVie-IRES-HygroR encoding VSV-P with intramolecular insertion of MERS-CoV M^{pro}. N-terminal glutamine was mutated to asparagine to knock-out one of the two cis-cleavage sites of M^{pro}. VSV-P with intramolecular insertion of MERS-CoV M^{pro} constitutes M^{pro}-On construct.

Plasmid 5: pLenti CMVie-IRES-BlastR encoding VSV-L polymerase gene.

Plasmid 6: pLenti CMVie-IRES-BlastR encoding VSV-L polymerase gene with a N-terminal tag of MERS-CoV M^{pro} and GFP, resulting in M^{pro}-Off construct.

Plasmid 7: E.coli MERS-CoV-Mpro expression plasmid with N-terminal His-tag and cleavable sequence for His-tag removal.

Sequences 1 to 8

Sequence 1-8: SARS-CoV-2 (NCBI: NC045512.2), SARS-CoV-1 (NCBI: AAP82976.1), MERS-CoV (NCBI: KF600615.1), HKU9 (NCBI: NC009021.1), NL63 (NCBI: YP010229075.1), 229E (NCBI: KF293666.1) and MHV (NCBI: NP045298.1) main proteases and poliovirus 3C-protease (NCBI: ACH87554.1) sequences as they were used in On, Off and virus constructs.

Files 1 to 3

File 1: Sequence identity distances table of alignment 5.

File 2: Sequence identity distances table of alignment 6.

File 3: Sequence identity distances table of alignment 7.
